# Magnetotherapy, alternative medicines, Hippocratic oath

**DOI:** 10.1186/1475-925X-7-1

**Published:** 2008-01-02

**Authors:** Max E Valentinuzzi

**Affiliations:** 1 Instituto Superior de Investigaciones Biológicas (INSIBIO), Tucumán, Argentina, Universidad Nacional de Tucumán (UNT), Tucumán, Argentina; 2 Consejo Nacional de Investigaciones Científicas y Técnicas (CONICET), Buenos Aires, Argentina

Not long ago, my wife had knee replacement surgery. Her surgery was successfully and skillfully carried out and, as expected, recovery went slow and rather painful. At dismissal, her surgeon, among several instructions, prescribed magneto-therapy for her pain. When I read it, I told him that the method should not be used because neither its effectiveness nor safety had yet been demonstrated. He looked at me in disbelief, as if saying, "*Who the heck are you to question my word?" *while adding, "*well, in burned subjects, it enhances good cell proliferation, but, if you do not want, it is fine with me"*. Hence, he condescendingly complied with my irreverent comment. The question, however, in a case like this, lies not on whether the patient wants or not the procedure, but rather settles on the much more important fact that **it must not be applied**.

Advocates say that therapy with magnets or pulsed magnetic fields is effective in reducing pain. However, I have seen no good studies demonstrating that. Conversely, there are good reports showing that such devices are ineffective, for example the recent double-blinded, placebo-controlled paper by Fernandez *et al *[1]. These authors concluded that pulse magnetic field therapy had no effect on pain.

And where is the evidence that such treatments are safe? Advocates argue that the treatments are without adverse effect. I am aware of several cases in which patients have apparently suffered from severe pain related to magneto-therapy after hip replacement or other orthopedic procedure. One might conjecture that heating of implants by the eddy currents induced in them by the varying magnetic fields might be a factor, in combination with the proximity of the implants to damaged and consequently sensitive tissues. Admittedly, my data are anecdotal and, to the best of my knowledge, there has been no systematic follow-up of patients treated with pulsed magnetic fields that might have uncovered any such effects had they occurred.

The possible uses of magnetic, and later on, electromagnetic fields have fascinated people for many years. Among the artifacts displayed at the Bakken Museum, in Minneapolis, MN, is an impressive solenoid coil from the 19^th ^Century, in which a patient stood while an electric current circulated through the coil. The patient's whole body was immersed in a magnetic field supposedly to accomplish some healing action. The attractiveness of such treatments might result from the placebo effect, or perhaps the certain degree of mystery surrounding them; the human being (and the human body, too) often act in funny unexpected strange ways [2].

A recent search identified more than 58,000 websites on magneto-therapy. One of them [3], says (after deleting a few irrelevant lines and even a few errors):

*Magneto-therapy is a safe physiatrist method in treatment of many diseases. Pulsed magneto-therapy *(PMT)*has had a long history in Europe, namely in the East and Middle Europe, whereas therapy effects of static magnetic fields were researched in the West. Modern PMF devices allow generating various frequencies, modulations, shape of impulses, exposure duration, etc. Frequencies used are between 1 and 100 Hz, magnetic flux density being up to 100 mT*.

*There are three established physical mechanisms through which pulsed magnetic fields interact with living matter: (1) magnetic induction; (2) magneto-mechanical effects; and (3) electronic interactions. Magnetic field exhibits the following activities: (1) vasodilatation; (2) analgesic action; (3) anti-inflammatory action; (4) spasmolytical activity; (5) healing acceleration; (6) anti-edematous activity*.

*It is known that PMF affects cellular level. PMF causes activation of enzymatic processes, activation of the metabolic transfers and functions of cellular membrane. Cellular respiration is activated in the exposed area. PMF produces positive changes in immunological condition of the patient, vasodilatation of the arterial part of capillaries, decreases blood coagulation*.

This is quite a list. The website describes some mechanisms of action of pulsed magnetic field therapy and refers to an international congress on the subject held in London in 1996. It even mentions clinical tests, simply describing their results as "excellent". Claims for such a medical panacea raise doubts and reservations. This skepticism is supported by recent articles and editorials in the conventional scientific literature. Finegold and Flamm state [4]:

"*Magnetic devices that are claimed to be therapeutic include magnetic bracelets, insoles, wrist and knee bands, back and neck braces, and even pillows and mattresses. Their annual sales are estimated at $300 millions in the United States and more than a billion dollars globally. They have been advertised to cure a vast array of ills, particularly pain. A Google search for the terms "magnetic + healing", omitting "MRI", yielded well over 20,000 pages, again, a spectacular claim. Many "controlled" experiments are suspect because it is difficult to blind subjects to the presence of a magnet*.

*For carpal tunnel syndrome pain, a double blind randomized study using magnet therapy ensured that magnets and shams were boxed individually so the treatments should not be identified. There was no statistical difference between the magnet and sham, yet both showed an improvement*.

*It is relevant referring to cost-benefit ratios in clinical practice that magnets, which are claimed to be therapeutic, have caused financial harm. Money spent on expensive and unproved magnet therapy might be better spent on evidence based medicine. More importantly, self treatment with magnets may result in an underlying medical condition being left untreated. Sadly, some advertisers even claim that magnets are effective for cancer treatment and for increasing longevity; not surprisingly, these claims are unsupported by data*.

*Magnets are touted by successful athletes, allowed to be widely advertised, and sold without restrictions, so it is not surprising that lay people think that claims of therapeutic efficacy are reasonable. However, even theoretically, magnet therapy seems unrealistic. If human tissue were affected by magnets, one would expect the massive fields generated by magnetic resonance imaging (MRI) to have profound effects. Yet the much higher magnetic fields of MRI show neither ill nor healing effects. Extraordinary claims demand extraordinary evidence. If there is any healing effect of magnets, it is apparently small since published research, both theoretical and experimental, is weighted heavily against any therapeutic benefit. Patients should be advised that magnet therapy has no proved benefits*.

More than 50 years ago my father, Máximo Valentinuzzi, carried out several studies trying to separate the weed from the good herb in magneto biology [5]; more recently I reviewed the field in historical perspective [6].

The broad rubrics of magneto biology and biomagnetism include well-established and scientifically respectable subjects such as the magnetocardiogram, magnetoencephalogram, magnetomyogram and stimulation of the brain using very intense magnetic field pulses. But magneto-therapy, particularly as it is described in numerous Websites, has a lot of quackery as well. Physicians prescribe magneto-therapy without enough knowledge, kinesiologists and physical therapists use magneto-therapy indiscriminately, medical insurance companies reimburse for such procedures (often inadequately, leaving patients responsible for costs for partially covered fees), and magneto-therapy equipment can be freely purchased on the Internet. Obviously, many sly people make money out of ignorance of the rest while endangering the health of innocent patients who, one way or another must pay the bills.

I do not categorically deny the possibility of beneficial effects of therapy using weak magnetic fields; perhaps, some do exist. My complaint is about the worldwide clinical use of electromagnetic fields without proper scientific proof and adequate trials. The list of beneficial effects that have been claimed for magnetic field therapy is so large that a whole laboratory could spend many years trying to prove them.

This situation contrasts notably with other areas of medical knowledge, such as the effects of vaccines, antibiotics, hormone treatments, beta blockers, contraceptive pills, aspirin, vitamins, cardiac pacemakers, implanted defibrillators, and the like, which have been subject to long and hard research, including investigations into probable side effects. Magneto-therapy, should it aspire to a similar acceptance, would face a similar period of rigorous study using standard methods of clinical research [7]. Companies selling such devices should sponsor adequate tests along the lines of those used for other medical devices. Besides, perhaps health agencies (either in the US or abroad) may view these as "alternative medicine" and do not demand the same level of proof that FDA demands for medical devices.

Caring for patients is the central objective of health care. The Hippocratic Oath, which is sworn by all physicians and forgotten by many, does not promise glory but stresses the obligation to care for patients [8]. But "caring for patients", in these days of high-technology medicine, is more than a matter of laying of hands by physicians, who by and large are ignorant of basic science and engineering, employing methods that are not well supported by good scientific studies. Close collaboration of physicians, biomedical engineers, and basic medical scientists is needed to help clarify the confusion and misuse surrounding the application of magnetic fields for medicine. It is time to move past the stage of amateur research and claims published on the Internet, and on to well controlled double-blinded studies with adequate engineering support and papers published in conventional scientific journals. While the prospects do not look good for a breakthrough in therapies using weak magnetic fields, perhaps something useful will be uncovered.

## References

[B1] Fernandez MI, Watson PJ, Rowbotham DJ (2007). Effect of pulsed magnetic field therapy on pain reported by human volunteers in a laboratory model of acute pain. British J Anaesthesia.

[B2] Valentinuzzi ME (1997). Del hechicero-médico al ingeniero biomédico (From the witch doctor to the biomedical engineer). Revista Federación Argentina Cardiología.

[B3] http://www.magnetotherapy.com/.

[B4] Finegold L, Flamm BL (2006). Magnet therapy: Extraordinary claims, but no proved benefits. British Medical J.

[B5] Valentinuzzi M, Alexander HS (1961). Magnetobiology and Complements of Magnetochemistry.

[B6] Valentinuzzi ME (2004). Magnetobiology: A historical view. IEEE/EMB Magazine.

[B7] Friedman LM, Furberg CD, DeMets DL (1998). Fundamentals of Clinical Trials.

[B8] Valentinuzzi ME (1996). Hippocrates' Oath. IEEE Technology & Society.

